# Trimetallic Fe-Zn-Mn (Oxy)Hydroxide-Enhanced Coffee Biochar for Simultaneous Phosphate and Ammonium Recovery and Recycling

**DOI:** 10.3390/nano15110849

**Published:** 2025-06-02

**Authors:** Diana Guaya, Jhuliana Campoverde, Camilo Piedra, Alexis Debut

**Affiliations:** 1 Departamento de Química, Universidad Técnica Particular de Loja, Loja 110107, Ecuador; 2 Escuela de Ingeniería Química, Universidad Técnica Particular de Loja, Loja 110107, Ecuador; 3 Centro de Nanociencia Nanotecnología, Universidad de las Fuerzas Armadas ESPE, Sangolquí 171103, Ecuador

**Keywords:** biochar, phosphate, ammonium, adsorption, circular economy

## Abstract

Excess phosphorus (P) and nitrogen (N) in wastewater contribute to eutrophication, driving the need for low–cost and sustainable recovery technologies. This study presents a novel adsorbent synthesized from spent coffee grounds biochar (CB) chemically modified with Mn^2+^/Zn^2+^/Fe^3+^ (oxy)hydroxide nanoparticles (CB–M) for simultaneous removal of phosphate and ammonium. Batch adsorption experiments using both synthetic solution and municipal wastewater were conducted to evaluate the material’s adsorption performance and practical applicability. Kinetic, isotherm, thermodynamic, and sequential extraction analyses revealed that CB–M achieved maximum phosphate adsorption capacities ranging from 42.6 to 72.0 mg PO_4_^3−^·g^−1^ across temperatures of 20–33 °C, reducing effluent phosphate concentrations to below 0.01 mg·L^−1^. Ammonium removal was moderate, with capacities ranging between 2.8 and 2.95 mg NH_4_^+^·g^−1^. Thermodynamic analysis indicated that phosphate adsorption was spontaneous and endothermic, dominated by inner–sphere complexation, while ammonium uptake occurred primarily through weaker, reversible ion exchange mechanisms. Sequential extraction showed over 70% of adsorbed phosphate was associated with Fe-Mn-Zn phases, indicating the potential for use as a slow–release fertilizer. The CB–M retained structural integrity and exhibited partial desorption, supporting its reusability for nutrient recovery. Compared to other biochars, CB–M demonstrated superior phosphate selectivity at a neutral–pH, avoided the use of hazardous metals, and transformed coffee waste into a multifunctional material for wastewater treatment and soil amendment. These findings underscore the potential of CB–M as a circular economy solution for nutrient recovery without introducing secondary contamination.

## 1. Introduction

Eutrophication is one of the most challenging environmental problems affecting surface water bodies worldwide. It is an ecological process, like natural aging, in which water bodies become increasingly enriched with essential nutrients, primarily nitrogen (N) and phosphorus (P), leading to increased primary productivity and photosynthesis [1]. This nutrient over-enrichment causes deleterious consequences such as harmful algal blooms, the proliferation of cyanobacteria and aquatic vegetation, oxygen depletion (hypoxia or anoxia), and decline in aquatic biodiversity. Consequently, eutrophication is recognized as a major driver of ecological degradation in both inland and coastal water bodies [2]. While eutrophication may occur naturally over geological timescales, anthropogenic eutrophication has been accelerated by human activities, particularly the excessive use of phosphate-containing detergents and nutrient-rich agricultural fertilizers [3].

Nitrogen is one of the most abundant elements in the biosphere and occurs in wastewater in both organic and inorganic forms, including ammonia (NH_4_), nitrite (NO_2_), and nitrate (NO_3_^−^) [4]. Among these, NH_4_ is frequently identified as a priority pollutant due to its high concentration in municipal, industrial, and aquaculture effluents [5]. Although nitrogen is essential for biological functions, its accumulation in aquatic environments poses substantial ecological risks and public concerns. Simultaneously, the global demand for ammonium continues to rise due to its widespread application in chemical fertilizers and industrial processes [6].

Phosphorus is a critical but non-renewable element, essential for agriculture, food security, and industrial production. It is obtained from phosphate rock, which contains 28–40% P_2_O_5_ by weight [7]. Approximately 77% of global phosphate reserves are concentrated in Morocco, China, and the United States, leading to geopolitical vulnerabilities and unequal access. Since 2014, the European Commission has recognized phosphorous as a critical raw material, underscoring its strategic importance [8]. Current projections estimate that economically viable phosphate reserves may be depleted by 2170, emphasizing the need for sustainable phosphorus recovery strategies [7]. Global demand is expected to continue rising due to population growth and its extensive use in fertilizers, food production, detergents, and metal processing.

To address these concerns, various technologies have been developed for phosphorus recovery from wastewater. Among them, struvite precipitation is widely studied and has the potential to meet 15–20% of the global phosphorus fertilizer demand [9]. However, its economic viability is limited to low phosphate concentrations. Electrochemical phosphate recovery methods have shown promise due to the elimination of chemical additives and operational flexibility [10]. Adsorption-based processes are particularly attractive because of their cost-effectiveness, operational simplicity, high efficiency, and potential for adsorbent regeneration [11]. Materials explored for the simultaneous removal of phosphate and ammonium removal included natural and engineered adsorbents such as biochar, zeolites [12], and metal-organic frameworks (MOFs) [13].

Biochar is a porous, carbon-rich material produced via pyrolysis of biomass under limited oxygen conditions. It is valued for its high surface area, chemical stability [14], and abundance of oxygen, nitrogen, and sulfur containing functional groups, which confer negative surface charge and facilitate cation adsorption [15]. Biochar can be produced from diverse organic feedstocks, including agricultural residues, municipal solid waste, food waste, and animal manure [16]. Spent coffee grounds (SGCs), a by-product of the coffee industry, are a particularly promising feedstock due to their high organic content and availability. SCGs are generated in quantities exceeding nine million tons annually, posing a major waste management challenge if not valorized properly [17]. The thermochemical conversion into biochar offers environmental benefits by promoting circular economy practices [18], reducing landfill volumes, and enabling resource recovery [19]. Furthermore, SCG-derived biochar has been studied for applications beyond wastewater treatment [20], including use as a renewable fuel, feedstock for bioethanol production, and a source of valuable antioxidants and nutrients [21].

To improve the adsorption capacity of biochar for phosphate, chemical modifications, particularly with metals, have been widely investigated [22]. The incorporation of Mn^2+^/Zn^2+^/Fe^3+^ (oxy)hydroxide nanoparticles has been reported to enhance biochar’s surface area, porosity, and density of active sites [23,24]. Fe^3+^ contributes magnetic properties and facilitates separation while promoting strong ligand exchange with phosphate [25]. Mn^2+^ enhances surface redox potential and functional group density, thereby improving adsorption performance [26]. Zn^2+^ has also demonstrated increased affinity for phosphate binding. Moreover, multi-metal modifications have been shown to enhance adsorption performance by up to five times compared to monometallic systems [27]. Despite these advances, the use of heavy metals in biochar modification raises concerns regarding environmental toxicity and the potential for secondary contamination upon reuse [17]. In contrast, Fe, Mn, and Zn are micronutrients essential for plant growth and are considered environmentally benign, supporting their suitability for post-use applications such as soil amendment [18,19,20].

Nevertheless, significant research gaps remain. To date, no studies have evaluated the combined use of Fe^3+^/Zn^2+^/Mn^2+^ -modified spent coffee ground biochar (CB–M) for the simultaneous removal of phosphate and ammonium. Moreover, most studies have focused on synthetic wastewater, whereas real effluent presents complex matrices of competing ions, fluctuating pH, and variable physicochemical properties, which can significantly impact adsorption efficiency. To address these gaps, this study aims to develop and characterize a novel trimetallic biochar (CB–M) derived from spent coffee grounds for the dual recovery of phosphate and ammonium from wastewater. The specific objectives are to: (i) synthesize a multifunctional biochar modified with Mn^2+^/Zn^2+^/Fe^3+^ (oxy)hydroxides; (ii) evaluate phosphate and ammonium adsorption performance in both synthetic and real wastewater; (iii) investigate the influence of pH on phosphate removal efficiency; (iv) model the adsorption kinetics, isotherms, and thermodynamic behavior; and (v) assess nutrient release and regeneration potential, evaluating CB–M’s feasibility as a slow-release fertilizer. By integrating waste valorization, nutrient recovery, and sustainable agriculture, this study proposes a circular economy approach that addresses environmental pollution and resource scarcity without introducing secondary contaminants.

## 2. Materials and Methods

### 2.1. Collection of Spent Coffee Ground Waste

Spent coffee grounds (SCGs) were pretreated with a 0.1 M sodium hydroxide solution to remove interfering agents such as cellulosic biomass and hemicellulose residues [28]. This alkaline treatment also enhances the surface area of the raw material (spent coffee grounds). A ratio of 100 mL NaOH per 25 g of spent coffee grounds was used, and the mixture was stirred for 60 min. Following this, the material was filtered, washed with distilled water to achieve a neutral pH, and oven-dried at 90 °C for 24 h.

### 2.2. Biochar Synthesis from Spent Coffee Grounds

The pretreated biomass was subjected to pyrolysis in a muffle furnace. Approximately 40 g of spent coffee grounds were placed in a ceramic crucible and carbonized at 600 °C. The resulting biochar (CB) was cooled to room temperature, sieved to a particle size ≤200 μm, and stored in sealed containers. The biochar yield was 30% and it was calculated using Equation (1):

(1) $$Yield\;(\%)=\frac{final\;CB\;weight}{inital\;SCG\;weight}\times 100\%$$

### 2.3. Biochar Activation

The metal-modified biochar (CB–M) was synthesized via a coprecipitation method, adapted from previous studies that successfully employed similar protocols for the deposition of metal hydroxide phases onto adsorbent surfaces for environmental remediation applications [23,29,30,31]. To enhance adsorption capacity of the base material, unmodified biochar (CB) was chemically modified using a mixed-metal chloride solution consisting of 0.1 M FeCl_3_, MnCl_2_, and ZnCl_2_. A solid-to-liquid ratio of 5 g of CB to 25 mL of metal solution was used. The pH of the suspension was adjusted to 7, a condition known to promote the precipitation of metal (oxy)hydroxide species. Under these near-neutral pH conditions and in the absence of thermal calcination, Fe^3+^, Zn^2+^, and Mn^2+^ ions are expected to form Fe(OH)_3_, Zn(OH)_2_, and Mn(OH)_2_, respectively, according to their solubility and aqueous speciation equilibria. The suspension was stirred at 30 °C for 4 h to facilitate metal incorporation and surface precipitation. Following the modification process, the biochar was thoroughly washed with distilled water to remove unbound ions and then dried at room temperature for 24 h. The final product was designated as metal-modified biochar (CB–M).

### 2.4. Physicochemical Characterization Methods

The chemical composition of CB–M was determined by X-ray fluorescence (XRF) using an S1-turbosd Hand-held XRF Analyzer (Bruker, Billerica, MA, USA). X-ray diffraction (XRD) patterns were obtained using a D8 Advance A25 diffractometer (Bruker, Karlsruhe, Germany), with Cu Kα radiation (λ = 0.1542 nm) at 40 kV and 40 mA over a 2θ range of 4–90°. Surface morphology was examined by scanning electron microscopy (SEM) with Tescan Mira 3 field emission SEM (Brno, Czech Republic). Surface area was assessed by single-point nitrogen adsorption with a Micrometrics Chemisorb 2720 analyzer (Norcross, GA, USA). Before BET analysis, samples were degassed using the Micromeritics Chemisorb 2720 system under a continuous N_2_ flow (25 mL/min), with a temperature ramp of 4 °C/min up to 200 °C, and held for 2 h to remove physically adsorbed species. A 36:64 N_2_:He volumetric mixture was used during analysis; helium acted as a non-adsorbing gas to determine the dead volume (cell free space), enhancing baseline correction and measurement accuracy. Each run used approximately 0.1000 g of sample. Fourier transform infrared (FTIR) spectra were collected using a Nicolet iS10 spectrometer (Jasco, Easton, MD, USA) in the range of 4000–500 cm^−1^. The point of zero charge (pH_PZC_) was determined using the pH drift method by suspending 0.1 g of biochar in 25 mL NaCl solution (0.01 or 0.05 M) across an initial pH range of 2–11. After 24 h, suspensions were centrifuged, and the final pH was recorded. All analyses were performed in triplicate.

### 2.5. Evaluation of Phosphate and Ammonium Adsorption

#### 2.5.1. Effect of pH on Phosphate and Ammonium Adsorption

To evaluate the influence of pH, 0.2 g of CB–M was added to 25 mL a 25 mg·L^−1^ phosphate and ammonium solution. The pH was adjusted between 2 and 11 using 0.1 N NaOH or HCl. After 24 h of agitation, suspensions were centrifuged and filtered through 0.45 µm syringe filters. Adsorption capacity was calculated using Equation (2):

(2) $$q_{e}=\frac{v\times (C_{o}-C_{e})}{w}$$

where $q_{e}$ is the equilibrium adsorption capacity (mg·g^−1^), v is the volume of phosphate and ammonium solution (L), $c_{o}$ and $c_{e}$ are the initial and equilibrium phosphate and ammonium concentration (mg·L^−1^ PO_4_^3−^), and w is the mass of the biochar CB–M (g).

#### 2.5.2. Phosphate and Ammonium Adsorption Kinetics

For kinetic studies, 0.2 g of CB–M was added to 25 mL of 25 mg·L^−1^ phosphate and ammonium solution at pH 7. Aliquots were collected at intervals from 5 s to 24 h, filtered, and analyzed. Adsorption capacity at time t ($q_{t}$) was calculated using Equation (3):

(3) $$q_{t}=\frac{v\times \;(C_{o}-C_{t})}{w}$$

Data were fitted to pseudo-first order (Equation (4)) and pseudo-second order (Equation (5)) kinetic models [32]:

(4) $$ln\left(q_{e}-q_{t}\right)={\ln q}_{e}-k_{1}t$$

(5) $$\frac{1}{q_{t}}=\frac{1}{k_{2}q_{e}^{2}}+\frac{t}{q_{e}}$$

where $k_{1}$ (h^−1^) is the pseudo-first-order rate constant, and $k_{2}$ (g·mg^−1^·h^−1^) is the pseudo-second-order rate constant.

The intraparticle diffusion model (Equation (6)), liquid film diffusion (Equation (7)) and particle diffusion (Equation (8)) models were also applied [33].

(6) $$q_{t}=k_{t\;}t^{1/2\;}+A$$

(7) $$\ln\left(1-\frac{q_{t}}{q_{e}}\right)=\frac{D_{f}c_{s}}{h\;r\;c_{z}}t$$

(8) $$-\ln\left(1-{\left(\frac{q_{t}}{q_{e}}\right)}^{2}\right)=\frac{2\pi^{2}D_{p}}{r^{2}}t$$

where k_t_ (mg·g^−1^·h^−1/2^) is the intraparticle diffusion rate constant and A (mg·g^−1^) represents the boundary layer thickness, D_f_ and D_p_ are diffusion coefficients (m^2^·s^−1^); $c_{s}$ (mg·L^−1^) and $c_{z}$ (mg·kg^−1^) are the adsorbate concentrations in solution and biochar (CB–M), respectively; r is the particle radius (3.7 × 10^−5^ m); t is the contact time (min); and h is the film thickness (1 × 10^−5^ m for a poorly stirred solution).

#### 2.5.3. Adsorption Isotherms

Isotherm studies were conducted at 293 K, 299 K, and 303 K with phosphate and ammonium concentrations ranging from 5 to 2000 mg·L^−1^ at pH 7. Adsorption data were fitted to the Langmuir model (Equation (9)) where adsorbate adsorption is confined to a molecular layer before a relative pressure of unity is reached [34]. r_L_ indicates the favorability of the adsorption process, 0 < r_L_ < 1 (Equation (10)). The Freundlich isotherm model (Equation (11)) outlines non-ideal adsorption on heterogeneous surfaces with active sites acting simultaneously with different energy [35]:

(9) $$\frac{c_{e}}{q_{e}}=\frac{c_{e}}{q_{m}}+\frac{1}{k_{L}q_{m}}$$

(10) $$r_{L}=\frac{1}{1+k_{L}c_{o}}$$

(11) $$\ln q_{e}=\ln k_{F}+\frac{1}{n}ln{\;c}_{e}$$

where $q_{m}$ is the maximum adsorption capacity (mg·g^−1^), $k_{L}$ is the Langmuir constant (L·mg^−1^), and $k_{F}$ and Freundlich constant (mg·g^−1^ PO_4_^3−^) and 1/n reflect the adsorption intensity or surface heterogeneity.

#### 2.5.4. Thermodynamic Analysis

Thermodynamic parameters including Gibbs free energy (ΔG°, kJ·mol^−1^), enthalpy (ΔH°, kJ·mol^−1^), and entropy (ΔS°, kJ·mol^−1^·K^−1^) were calculated using the van’t Hoff approach by using Equations (12) and (13) [36]:

(12) $$∆G{}^{\circ}=-RTlnk_{c}$$

(13) $$\ln\left(\frac{q_{e}}{c_{e}}\right)=\frac{∆S{}^{\circ}}{R}-\frac{∆H{}^{\circ}}{RT}$$

where $k_{c}$ is the equilibrium constant of the adsorption process (Equation (14)), R is the universal gas constant (8.314 J·mol^−1^·K^−1^), and T is the absolute temperature in Kelvin (K). The equilibrium constant $k_{c}$, was converted to dimensionless form by multiplying $k_{L}$ by the molecular weight of the adsorbate (g·mol^−1^), by 1000 and 55.5 [37]:

(14) $$k_{c}=k_{L}\times M_{w}\times 1000\times 55.5$$

#### 2.5.5. Biochar Regeneration

To assess reusability, CB–M saturated with phosphate and ammonium solution (25 mg·L^−1^) was washed, dried, and immersed in 0.1 M NaHCO_3_ with agitation for 24 h. Desorption capacity was calculated using Equation (15):

(15) $$q_{\mathrm{des}}=\frac{c_{e}\times v}{m}$$

where q_des_ is the desorption capacity (mg·g^−1^), $c_{e}$ is the phosphate concentration in the supernatant, v is the volume (L), and m is the biochar CB–M mass (g).

#### 2.5.6. Phosphate Fractionation

Phosphate-loaded CB–M was sequentially extracted to identify retention mechanisms. Labile phosphate was extracted using two treatments with 20 mL of 1 M ammonium chloride (NH_4_Cl). Metal-associated phosphate was extracted with 0.1 M NaOH (pH 11) and 1 M NaCl (pH 7). Alkaline earth-associated phosphate was removed using two treatments with 0.5 M HCl. Residual phosphate was determined by mass balance.

### 2.6. Application to Real Wastewater

Municipal wastewater effluent was collected from a treatment facility in Loja City, Ecuador. Physicochemical parameters (pH, turbidity, sulphate, nitrate, hardness, chloride, total solids, biochemical oxygen demand (BOD_5_), fluoride, cyanide, ammonia nitrogen, total ammonia, nitrites, bicarbonates, phosphate, and total phosphorus) were analyzed by an accredited laboratory (details in Supplementary Material). One liter of wastewater was treated with 0.125 g·L^−1^ under laboratory conditions (20.6 °C, 24 h). After treatment, the supernatant was separated by centrifugation and filtration, and residual phosphate and ammonium concentrations were determined to calculate removal efficiency.

## 3. Results and Discussion

### 3.1. Physicochemical Properties of Biochar

The chemical composition of the unmodified biochar (CB) and metal-modified biochar (CB–M) exhibited notable differences, as summarized in Table 1. The unmodified biochar (CB) primarily consisted of SiO_2_, P_2_O_5_, K_2_O, and CaO, with trace amounts of Fe_2_O_3_. These oxides are commonly found in biochar derived from lignocellulosic biomass such as spent coffee grounds, which are naturally rich in potassium and calcium.

Following modification with Mn^2+^, Zn^2+^, and Fe^3+^, CB–M showed a substantial increase in Fe_2_O_3_ (25.3%) and ZnO (63.9%), confirming the successful incorporation of these metals’ oxides into the biochar matrix. MnO (1.6%) was also detected, although at lower levels, indicating less efficient retention. The preferential incorporation of iron (Fe^3+^) and zinc (Zn^2+^) may be attributed to their stronger affinity for oxygen-containing surface functional groups, facilitating the formation of stable complexes. Prior studies have shown that Fe^3+^ and Zn^2+^ tend to form preferential and durable metal–oxygen bonds that enhances surface binding. The enhanced affinity of iron and zinc is explained by their smaller ionic radii (Fe^3+^ ≈ 65 pm, Zn^2+^ ≈ 74 pm) and higher charge densities compared to Mn^2+^ (≈83 pm), which facilitate stronger electrostatic interactions with functional groups (e.g., –OH, –COOH). Their higher hydration energies, Fe^3+^ (≈−4490 kJ/mol); Zn^2+^ (≈−2046 kJ/mol), compared to Mn^2+^ (≈−1840 kJ/mol); also promote the formation of stable surface complexes [38].

The reduction in K_2_O content observed in CB–M may result from ion exchange during metal impregnation, where potassium ions were replaced by Fe^3+^, Zn^2+^, and Mn^2+^, potentially generating new active adsorption sites.

**Figure 1:**
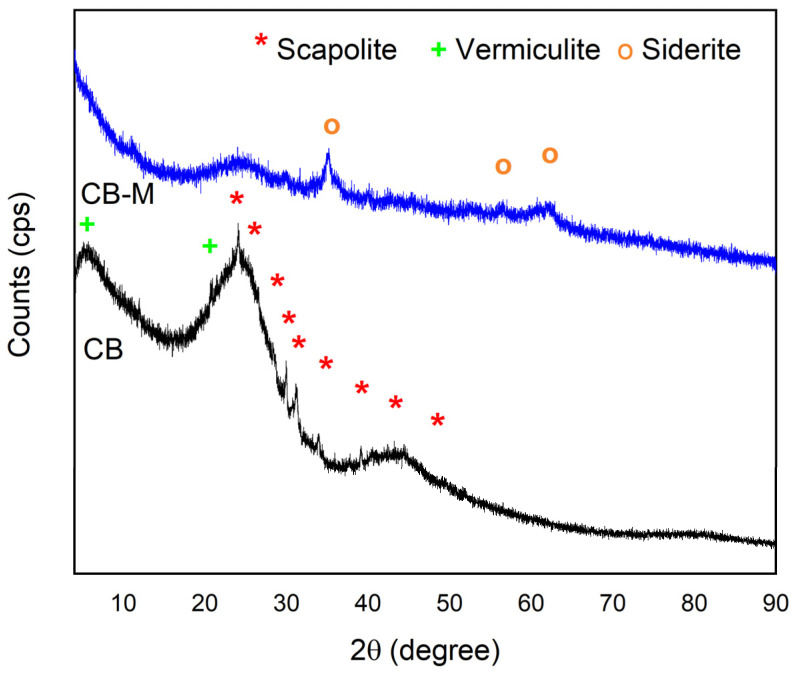
XRD patterns of spent coffee grounds biochar (CB) and its metal-modified form (CB–M).

The XRD patterns of the unmodified biochar (CB) and the metal-modified biochar (CB–M) further confirmed structural modification (Figure 1). Both samples displayed broad diffraction peaks in the 20–30° (2θ) range, characteristics of partially carbonized biochar. The diffraction pattern of CB showed peaks, and they were compared with the standard diffraction patterns from the Crystallography Open Database. In the unmodified biochar CB, diffraction peaks at 2θ values of 24.1°, 26.0°, 28.1°, 29.9°, 31.2°, 33.9°, 39.9°, 43°, and 47°, which correspond to scapolite (Ref. No. 9010511), indexed to the tetragonal crystal system (space group I 4/m), with unit cell dimensions a = b = 12.2 Å, c = 7.4 Å. Additionally, peaks at 5° and 21.5° were attributed to vermiculite (Ref. No. 9016797), a common clay mineral identified in biochars derived from spent coffee grounds, with triclinic symmetry (space group P 1), and unit cell parameters a = 5.3 Å, b = 9.3 Å and c = 14.3 Å. In the CB–M sample, scapolite remained the dominant crystalline phase; however, new peaks at 2θ: 35°, 56°, and 62° appeared, corresponding to siderite (Ref. No. 9014944; FeCO_3_), a trigonal mineral with hexagonal axes (space group R 3-c) with unit cell parameters a = b = 4.5 Å, and c = 13.3 Å. The presence of siderite suggests that iron precipitated as carbonate due to reaction with CO_2_ from the atmospheric or the aqueous source during the synthesis consistent with previous reported on Fe-modified biochars. A contraction in the d-spacing of the scapolite hkl (002) reflection was observed, shifting from 3.68 Å (2θ = 24.1°) in CB shifted to 3.58 Å (2θ = 24.8°), indicating lattice distortion due to Fe^3+^ and Zn^2+^ substitution or intercalation. These changes are attributed to the smaller ionic sizes and higher charge densities of the introduced metal ions (Fe^3+^ ~65 pm and Zn^2+^ ~74 pm), which induce tighter packing and stronger bonding, reducing interplanar distances. Although XRD patterns confirmed the formation of FeCO_3_ (siderite), distinct and well-resolved diffraction patterns corresponding to Fe(OH)_3_, Zn(OH)_2_, and Mn(OH)_2_ were not detected. This absence is likely due to low crystallinity, amorphous nature, or low concentrations of these phases, which fall below the detection limit of the XRD technique. However, the increased background signal observed in the CB–M diffractogram suggests the presence of amorphous or poorly crystalline materials, consistent with the formation of surface-deposited metal (oxy)hydroxides such as Fe(OH)_3_ (s), Zn(OH)_2_ (s), and Mn(OH)_2_ (s) during the pH-controlled impregnation process. The synthesis was conducted at near-neutral pH ~ 7 through the addition of NaOH and without any post-synthesis thermal treatment, conditions well-documented to promote the precipitation of metal hydroxides. This methodology has been widely applied in the literature to generate surface-bound hydroxide phases on biochar and other porous supports [23,29,30,31]. Under these conditions, Fe^3+^ and Zn^2+^ readily precipitate and adhere to the biochar surface, as further supported by Medusa speciation diagrams (Supplementary Figure S1). In contrast, Mn^2+^ remains more soluble at this pH and is, therefore, more susceptible to partial loss during subsequent washing steps. While all three metals can form solid hydroxide phases [M(OH) (s)], they exist in dynamic equilibrium with their aqueous ionic counterparts [M^+^ (aq) and OH^−1^ (aq)]. Their retention on the biochar surface is governed by their relative solubility, coordination strength, and the surface functional groups available for binding [38].

**Figure 2:**
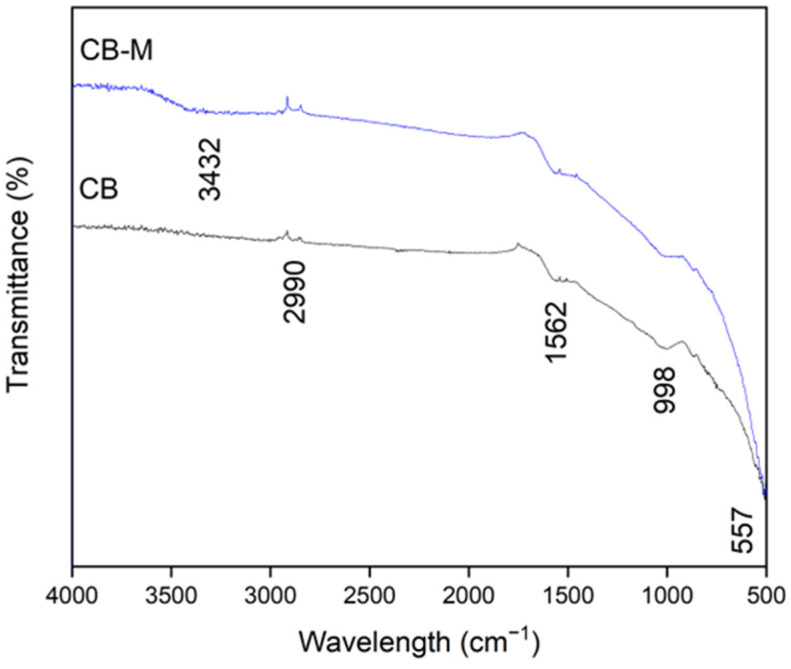
FTIR spectra of spent coffee grounds biochar (CB) and its metal-modified form (CB–M).

FTIR analysis provided additional evidence of chemical groups existent on both unmodified biochar (CB) and metal-modified biochar (CB–M) (Figure 2). In CB, bands were observed at 2990, 1562, 998, and 557 cm^−1^. The band at ~2990 cm^−1^ corresponds to aliphatic C–H stretching from methyl and methylene groups, that residues often retained from original biomass [39]. The band at ~1562 cm^−1^ is attributed to C=C aromatic ring stretching or carboxylate asymmetric stretching vibrations (–COO^−^), indicative of lignin-derived structures [40]. The peak at ~998 cm^−1^ is associated with the presence of inorganic functional groups (Si-O) detected in biomass-derived chars with trace minerals as well as those associated with P-O-P bonds [41]. The absorption band at ~557 cm^−1^ may correspond to bending vibrations of mineral phases or weak metal–oxygen bonds inherent to the original biomass [42]. In CB–M, changes in peak intensity and position reflected metal interactions with surface functional groups of biochar; however, the primary organic framework of the original is retained. Notably, the broad O–H stretching band near 3432 cm^−1^ intensified, indicating enhanced hydroxylation and adsorbed water due to the presence of metal hydroxides facilitating hydrogen bonding. The shifts observed in the ~998 cm^−1^ and ~557 cm^−1^ bands imply the coordination between metal ion and functional groups, consistent with new metal–O bonds and supported by XRD-detected formation of siderite [7]. The FTIR spectral features observed in CB–M, particularly the intensified O–H stretching band, the persistence of stable aromatic structures, and the appearance of metal–oxygen coordination bands, are consistent with those reported for metal-loaded biochars derived from lignocellulosic residues [43]. These characteristics support the presence of surface-bound metal (oxy)hydroxide species, as previously discussed. Notably, the persistence of the characteristic band near 1562 cm^−1^, attributed to aromatic ring stretching C=C or asymmetric –COO^−^ stretching confirms that the core aromatic carbon framework of the biochar remained structurally intact following metal modification. This observation is in agreement with previous studies on metal-functionalized biochars synthesized from spent coffee grounds [44].

**Figure 3:**
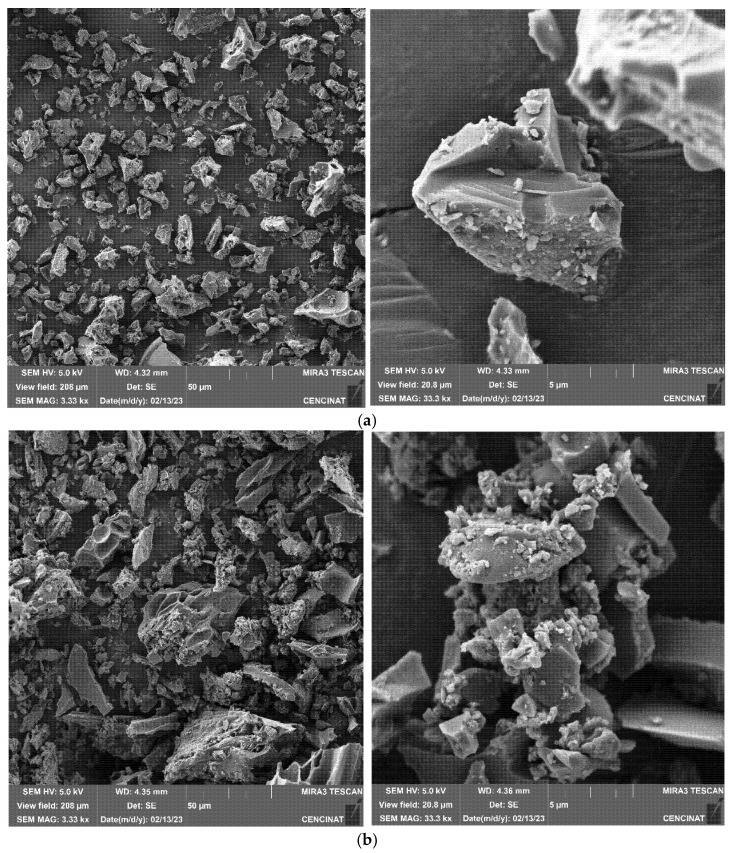
SEM micrographs of (**a**) unmodified CB and (**b**) metal-modified CB–M biochar.

Scanning electron micrographs revealed significant morphological differences following modification (Figure 3). The surface of unmodified CB was smooth and featured macropores (1–10 μm) formed via pyrolysis from the volatilization of organic matter, which creates voids and channels, characteristics of biochar from lignocellulosic biomass. In contrast, CB–M displayed heterogeneous surface nanoaggregates (50–120 nm) on the biochar surface (Figure 3b), associated with precipitated metal hydroxide precipitates of Mn^2+^, Zn^2+^, and Fe^3+^, partially blocking macropores but also contributing to a more complex spore structure due to the formation of micro- and mesopores. The appearance of particle clusters on the carbon matrix have been reported in other studies on metal-loaded biochars. The specific surface area of unmodified biochar (CB) was determined to be 3 m^2^·g^−1^. After modification with Mn^2+^, Zn^2+^, and Fe^3+^ (oxy)hydroxide phases, the surface area of the resulting material (CB–M) increased to 22 m^2^·g^−1^, representing a seven-fold enhancement. This increase reflects the formation of hierarchical porosity and partial structural activation, which introduces new accessible surface features despite the deposition of metal nanostructures. The incorporation of Mn^2+^, Zn^2+^, and Fe^3+^ (oxy)hydroxides likely contributes to both the creation of additional reactive sites and partial pore blockage, an expected trade-off that may limit surface area expansion but enhances functional surface chemistry. Although the surface area of CB–M remains modest compared to highly engineered porous materials, it falls within the range commonly reported for spent coffee ground biochars depending on the activation strategy, pyrolysis temperature, and post-treatment protocol. For instance, coffee grounds pyrolyzed at 500 °C and activated with NaOH achieved a surface area of 46.3 m^2^·g^−1^ [45], while treatment with NaHCO_3_ at 300 °C increased the surface area from 0.07 to 42.1 m^2^·g^−1^ [46]. Similarly, NaOH activation of spent coffee ground biochar pyrolyzed at 500 °C raised the surface area from 3.6 to 116.6 m^2^·g^−1^ [47], and acid modification of spent coffee ground biochar increased the surface area from 2.4 to 82.1 m^2^·g^−1^ [48].

In metal-based modifications, even higher surface areas have been reported. For example, iron oxide-modified spent coffee grounds biochar exhibited surface areas ranging from 550.3 m^2^·g^−1^ at 400 °C to 879.6 m^2^·g^−1^ at 800 °C [49]. Another study showed that Fe (III)- and KOH-modified biochar at 700 °C exhibited a surface area of 59.2 m^2^·g^−1^ compared to 13.1 m^2^·g^−1^ for the unmodified sample [50]. Scanning electron microscopy (SEM) further revealed the formation of microcracks near the metal deposits, indicative of localized structural stress, which is consistent with scapolite lattice distortion and siderite (FeCO_3_) formation identified by XRD. These structural features align with observations by Yang et al. (2023), who reported that biochars produced via slow pyrolysis develop more pronounced porosity and surface cracking than those produced by torrefaction, due to greater volatile loss and matrix reorganization during thermal treatment [51]. Overall, while CB–M does not exhibit the highest surface area among biochars reported in the literature, its adsorption performance is primarily governed by the presence of reactive surface-bound metal (oxy)hydroxide phases. This highlights that surface chemistry, rather than surface area alone, plays the dominant role in controlling nutrient adsorption efficiency in this system.

### 3.2. Influence of pH on Phosphate and Ammonium Adsorption

**Figure 4:**
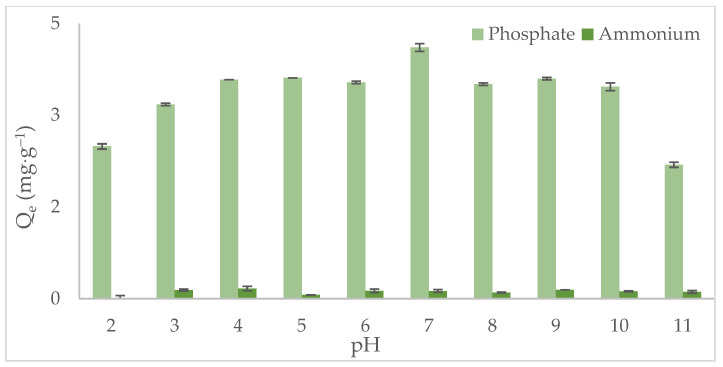
Phosphate and ammonium adsorption capacities as a function of pH onto metal-modified spent coffee grounds biochar (CB–M).

The adsorption behavior of phosphate and ammonium ions onto the metal-modified biochar (CB–M) was evaluated as a function of pH (Figure 4). The point of zero charge (pH_PZC_) of CB–M was determined to be 7.0 ± 0.2, indicating that the surface of the metal-modified biochar is electrically neutral at this pH. Below this value, the biochar surface becomes positively charged, favoring the adsorption of anionic species such as phosphate. Conversely, above the pH_PZC_, the surface acquires a net negative charge, which enhances the adsorption of cationic species like ammonium. Phosphate adsorption was most favorable at pH values slightly below 7, where the dominant phosphate species (e.g., H_2_PO_4_^−^, HPO_4_^2−^) interact electrostatically with the positively charged surface [23]. Under strongly acidic conditions (around pK_a1_ = 2.7), the neutral form of phosphate predominates (e.g., H_3_PO_4_), reducing adsorption capacity due to limited electrostatic attraction. As pH increases above the pH_PZC_, the biochar surface becomes more negatively charged, reducing phosphate uptake because of electrostatic repulsion [24]. Ammonium (NH_4+_), in contrast, remains cationic over the entire pH range evaluated, and its adsorption increased at a pH value above 7, where the negatively charged CB–M surface enhances electrostatic attraction. At pH values below the pH_PZC_, repulsion between the positively charged surface and ammonium ions leads to decreased adsorption [52]. Overall, both phosphate and ammonium adsorption processes are highly pH-dependent, driven by interplay between the biochar’s surface charge and the ionic speciation of the adsorbates [53]. Maximum adsorption for both species was observed at pH 7, suggesting that in addition to electrostatic interactions, specific surface binding mechanisms, such as complexation and ion exchange, also play a significant role. These findings are consistent with previous reports highlighting the critical influence of pH on surface charge modulation, adsorbate chemical speciation, and binding site reactivity in metal-modified biochar.

### 3.3. Kinetic of Phosphate and Ammonium Adsorption

**Figure 5:**
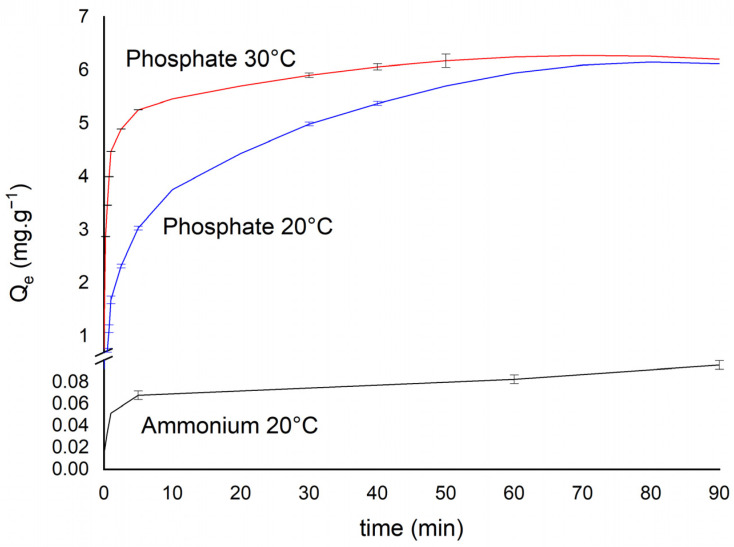
Kinetics of phosphate and ammonium adsorption onto metal-modified spent coffee grounds biochar (CB–M).

Phosphate adsorption onto CB–M reached equilibrium within approximately 40 min, while ammonium equilibrated in just 10 min (Figure 5). Although the ammonium was adsorbed more rapidly, the maximum adsorption capacity of CB–M was significantly higher for phosphate. This indicates that phosphate exhibits a stronger interaction with the active sites of the biochar, while ammonium is rapidly adsorbed but quickly reaches saturation due to limited binding sites. Temperature also influenced phosphate adsorption. At 30 °C, phosphate uptake reached approximately 6 mg·g^−1^ within the first 30 min, compared to 5 mg·g^−1^ at 20 °C. This temperature dependence suggests that phosphate adsorption is at least partially endothermic, consistent with previous findings. These results demonstrate that CB–M effectively removes phosphate in a relatively short time, particularly at elevated temperatures, whereas ammonium exhibits faster kinetic but lower capacity. Compared to conventional biochars, CB–M demonstrate superior kinetic performance when evaluated against other biochars reported in recent literature. For instance, Xi et al. (2022) reported that a magnesium-modified biochar bead system reached equilibrium for phosphate and ammonium adsorption in approximately five hours [54]. Similarly, Feng et al. (2022) noted that calcium-based activated biochar achieved equilibrium phosphate and ammonium ions adsorption in approximately 8 h [55]. The rapid kinetics and higher phosphate uptake of CB–M thus underscore its potential as an effective material for nutrient recovery in wastewater treatment applications.

Additionally, the homogenous diffusion models considered phosphate and ammonium sorption onto CB–M may be limited by diffusion through the boundary layer or internal pores. To further assess diffusion mechanisms, both film diffusion model if diffusion occurred in the film phase (D_f_, m^2^·s^−1^) governing the adsorption rate (Equation (7)) and particle diffusion when the rate of adsorption is controlled by CB–M particle diffusion model (Equation (8)) [56].

The maximum phosphate adsorption capacity was 6.38 mg·g^−1^, significantly higher than the 0.12 mg·g^−1^ recorded for ammonium, confirming a stronger affinity for phosphate (Table 2). The pseudo-first order model yielded moderate correlation coefficients (R^2^ = 0.98 for phosphate and 0.90 for ammonium), suggesting partial involvement of multilayer adsorption or physisorption mechanisms. However, the pseudo-second order model provided the best fit for both ions (R^2^ = 1.00), indicating that chemisorption or surface-specific binding or chemisorption is the rate-limiting step of adsorption process. Interestingly, the pseudo-second order rate constant k_2_ was significantly higher for ammonium (43.00 h^−1^) than for phosphate (1.95 h^−1^), suggesting that ammonium is adsorbed more rapidly, via physical mechanisms such as ion exchange. The faster equilibrium supports the hypothesis of limited available sites for ammonium, quickly becoming saturated. Conversely, phosphate interactions proceed more slowly due to the chemical mechanisms or specific adsorption due to the larger number of high-affinity binding, such as ligand exchange with metal (oxy)hydroxide groups [57]. Ammonium adsorption, primarily driven by cation-exchange, is less efficient than the ligand exchange and inner-sphere complexation mechanisms responsible for phosphate uptake [58]. These contrasting mechanisms underscore CB–M’s selectivity for phosphate. The intraparticle diffusion model also showed strong correlation (R^2^ ≥ 0.96) for phosphate, revealing two adsorption stages: an initial boundary layer diffusion phase followed by slower intraparticle diffusion stage as external sites are occupied, confirming significant pore diffusion, involving both microporous and mesoporous structures. Ammonium exhibited lower R^2^ values, suggesting a lesser contribution from pore diffusion. These findings are consistent with those by Feng et al. (2022), who reported similar multi-step intraparticle diffusion pathways for both phosphate and ammonium ions on calcium alginate–biochar composite [55].

### 3.4. Phosphate and Ammonium Adsorption Isotherm and Thermodynamic Analysis

The Langmuir model provided maximum phosphate adsorption capacities (q_m_) that increased with temperature, ranging from 42.6 mg·g^−1^ at 20 °C, to 57.8 mg·g^−1^ at 26 °C, and 72.0 mg·g^−1^ at 33 °C (Table 3). In contrast, ammonium adsorption capacities were lower, ranging from 2.79 mg·g^−1^, 2.84, to 2.95 mg·g^−1^ across the same temperatures. The high correlation coefficients (R^2^ > 0.92) for phosphate suggest that adsorption proceeds via monolayer chemisorption on homogeneous active sites, associated with surface metal (oxy)hydroxide phases on CB–M. The increase in capacity with temperature confirms that phosphate adsorption is endothermic, benefiting from enhanced molecular mobility and increased interaction with reactive sites. The Freundlich model also described phosphate adsorption well (R^2^ ≈ 0.83–0.86), indicating contribution from physical interactions and surface heterogeneity. In contrast, ammonium adsorption was better described by the Freundlich model (R^2^ up to 0.95), suggesting that it proceeds through weaker, non-specific mechanisms such as ion exchange and electrostatic attraction. The Freundlich constants (0.59 ≤ 1/n ≤ 0.74) support favorable heterogenous adsorption. These differences are attributed to the distinct chemical nature of the adsorbates: phosphate tends to form stronger inner-sphere complexes with surface metal groups via complexation and ligand exchange, whereas ammonium is adsorbed primarily through weaker physisorption mechanisms, including electrostatic attraction and cation exchange. These latter interactions are inherently less robust, resulting in lower adsorption strength and capacity. Notably, phosphate removal by CB–M is predominantly governed by chemisorption onto metal (oxy)hydroxide functional groups, rather than being driven solely by surface area. Consequently, the enhanced adsorption efficiency of CB–M is attributed to surface functionalization, supporting the critical role of surface chemistry in achieving high phosphate retention. Therefore, CB–M exhibits a significantly higher affinity for phosphate than for ammonium, making it especially suitable for nutrient recovery applications. These findings align with previous studies. Biswas et. al. (2024) reported that Mg-doped biochar exhibited a higher affinity for phosphate than for ammonium, with the Langmuir model best described phosphate adsorption (R^2^ > 0.93) [59], consistent with the results for CB–M, which also showed R^2^ > 0.92 for phosphate adsorption and increased capacity with temperature, confirming endothermic chemisorption mechanisms. However, adsorption behavior can vary depending on whether ions are adsorbed individually or in a binary system. Similarly, Takaya et al. (2016) found that phosphate adsorption onto waste-derived biochar followed the Freundlich model, while ammonium adhered better to Langmuir behavior, though attributed to heterogeneous ion exchange processes [60]. Our findings agree with these results: phosphate showed a higher capacity and Langmuir fitting, while ammonium was better explained by Freundlich, supporting CB–M’s selectivity toward phosphate.

Table 4 shows the thermodynamic parameters for both phosphate and ammonium adsorption onto CB–M calculated at 293.15 K, 299.15 K, and 306.15 K. Phosphate adsorption exhibited a negative value of Gibbs free energy (ΔG°) at all temperatures [61,62], confirming spontaneity. Additionally, the more negative ΔG° with increasing temperature suggests improved adsorption efficiency at higher temperatures [63]. The large positive enthalpy change (ΔH° 1450 kJ·mol^−1^) confirms the endothermic nature of the process, likely governed by complexation and ligand change interaction, due to improved molecular mobility and diffusion into the porous structure [64].

In contrast, ammonium adsorption showed less negative ΔG° values and small negative ΔS° −4 kJ·mol^−1^, suggesting lower randomness at the solid–liquid interface [29]. The lower enthalpy changes to weaker physical interactions such as electrostatic interactions [65] and ion exchange rather than strong chemical bonding [66]. Overall, the thermodynamic data confirm that phosphate adsorption onto CB–M is spontaneous and endothermic, driven by chemisorption mechanisms [67]. Conversely, ammonium adsorption is thermodynamically less favorable, dominated by physisorption and ion exchange [65].

**Figure 6:**
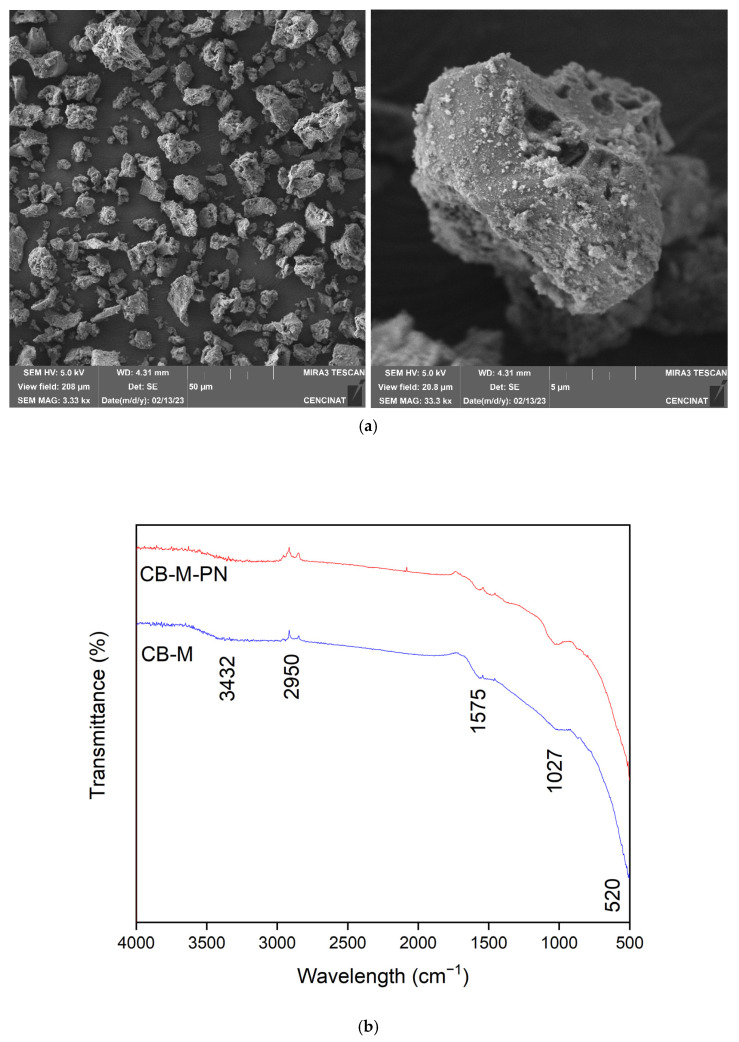
(**a**) SEM micrography and (**b**) FTIR spectra of CB–M-P-N after phosphate and ammonium adsorption.

SEM analysis (Figure 6a) revealed significant morphological changes in CB–M after phosphate and ammonium adsorption. The surface exhibited an increase in roughness and discrete particulate deposits, corresponding to adsorbed phosphate complexes [55]. These particles, ranging from 100 nm to 1 μm suggest phosphate complexation with metal (oxy)hydroxide sites through the formation of inner-sphere surface complexes, consistent with chemisorption mechanisms. The FTIR spectra (Figure 6b) exhibited an intensified O–H stretching band near 3432 cm^−1^ after adsorption, indicating the functional presence of surface-bound (oxy)hydroxide groups. This enhancement is attributed to increased hydrogen bonding interactions, likely arising from the coordination of hydrated phosphate or ammonium species with hydroxyl groups. Shifts in the 1562 cm^−1^ band (carboxylate or aromatic C=C stretching) suggest specific interactions with surface functional groups. New bands between 900 and 1200 cm^−1^ confirm P–O stretching vibrations, validating phosphate binding. In contrast, ammonium-induced spectral changes were subtle, consistent with weaker physisorption. These results support the proposed mechanisms: phosphate adsorption occurred via strong chemisorption as reported in other biochar materials [68]. Together, the SEM and FTIR results confirm that phosphate adsorption is driven by strong chemisorption, while ammonium interacts through reversible physical mechanisms, confirming CB–M as a dual-function adsorbent for nutrient (phosphate and ammonium) recovery from wastewater.

### 3.5. Extraction of Phosphate Fractions from Phosphate-Loaded Biochar

The distribution of phosphate among different chemical fractions labile, metal-associated, alkaline earth metal-associated, and residual, following sequential extraction is presented in Table 5. The predominance of the metal-bound fraction (Fe–Mn–Zn) accounting for approximately 72.1% of total adsorbed phosphate, suggest that the primary adsorption mechanism involves inner-sphere complexation with surface metal (oxy)hydroxide groups on CB–M. This supports chemisorption as the dominant mechanism governing phosphate retention. The labile fraction, representing 17.1% of the total adsorbed phosphate, indicates that a moderate portion of phosphate is weakly bound, through electrostatic interactions or ion exchange. This fraction is considered readily exchangeable and potentially bioavailable, making it of agronomic interest for short-term nutrient release if the spent biochar is repurposed as a soil amendment [3]. A smaller fraction 6.3% of phosphate was associated with alkaline earth metals (e.g., Ca^2+^, Mg^2+^), indicating a minor contribution from coordination with divalent cations. The residual fraction was the lowest, at 2.7%, suggesting minimal fixation into stable organic–mineral matrices or incorporation into recalcitrant phosphate compounds. These findings reinforce the high phosphate retention capacity previously observed and demonstrate the potential dual functionality of CB–M as both a wastewater adsorbent and a secondary phosphorous source for agricultural reuse.

### 3.6. Evaluation of Biochar Regeneration Capacity

**Figure 7:**
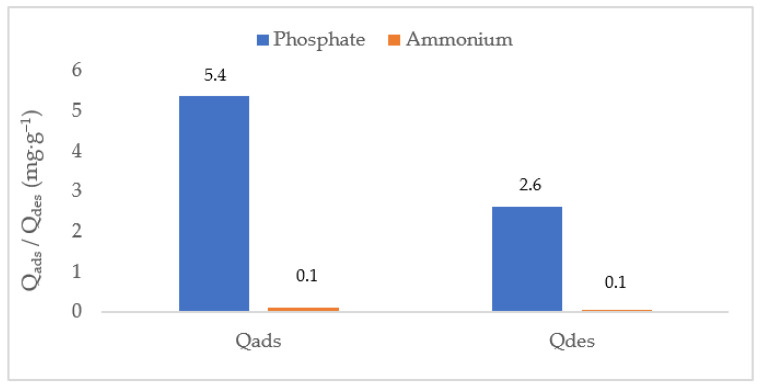
Desorption capacity of phosphate and ammonium from loaded metal-modified spent coffee grounds biochar (CB–M-P-N).

The regeneration potential of CB–M was assessed through desorption experiments for both phosphate and ammonium, as shown in Figure 7. The desorption capacity for phosphate was 2.2%, while that for ammonium was slightly lower, at 1.9%. These low desorption efficiencies confirm the irreversible nature of phosphate binding, due to inner-sphere complexation with Fe^3+^, Mn^2+^, and Zn^2+^ (oxy)hydroxide phases, which are resistant to mild desorbing agents NaHCO_3_. In contrast, ammonium desorption, though similarly limited, reflects its weaker interactions with the biochar surface, principally through electrostatic attraction and cation exchange mechanisms. The slightly greater desorption observed for ammonium supports this hypothesis and confirms its more reversible nature. Despite the low regeneration efficiencies under tested conditions, the nutrient-loaded CB–M retains its fertilization potential and may be repurposed as a slow-release fertilizer, thereby contributing to a circular nutrient economy. These results underscore CB–M’s value not only as an efficient adsorbent for nutrient recovery from wastewater but also as a resource-efficient amendment for sustainable agricultural applications.

In addition to evaluating phosphate desorption performance, the potential leaching of incorporated metals (Fe, Zn, Mn) from CB–M is recognized as a critical aspect concerning environmental safety and long-term material reuse. Although dedicated leaching assays were not conducted in this study, indirect evidence suggests minimal metal release under the applied conditions. This inference aligns with the known chemical behavior of Fe(OH)_3_ and Zn(OH)_2_, which exhibit high coordination stability and low aqueous solubility at near-neutral to mildly alkaline pH, thereby favoring their retention on the biochar surface. In contrast, Mn^2+^ species are more mobile under similar pH conditions and may exhibit a higher propensity for leaching. While this potential release could not be experimentally confirmed within the scope of this study, it agrees with previous reports highlighting the relative lability of Mn-based sorbents in aqueous environments [69]. To fully assess the environmental stability of CB–M, future investigations should include quantitative metal leaching analyses under variable pH conditions, ionic strengths, and through multiple regeneration cycles.

### 3.7. Phosphate and Ammonium Removal from Real Wastewater Using CB–M

The adsorption performance of CB–M was evaluated using real municipal wastewater. CB–M exhibited a high phosphate adsorption capacity of 30.16 mg·g^−1^, reducing the phosphate concentration in the treated effluent to below 0.01 mg·L^−1^, thereby meeting U.S. environmental discharge standards. This high removal efficiency underscores the strong affinity of CB–M’s active sites for phosphate species. In contrast, ammonium removal was less efficient, with an adsorption capacity of 1.84 mg·g^−1^ and a final effluent concentration of 18.87 mg·L^−1^. The lower ammonium adsorption efficiency is attributed to the complex composition of real wastewater, which contains a variety of co-existing ions, including sulphate (SO_4_^2−^), nitrate (NO_3_^−^), chloride (Cl^−^), fluoride (F^−^), and bicarbonate (HCO_3_^−^). These anions can compete with phosphate and ammonium for electrostatic and ion-exchange binding sites on the biochar surface [58]. Several previous studies have demonstrated that these co-existing species significantly reduce nutrient adsorption capacities. The potential interference of common anions such as sulfate (SO_4_^2−^), nitrate (NO_3_^−^), and chloride (Cl^−^) on phosphate and ammonium adsorption has been previously studied individually in synthetic and real urban wastewater using metal-modified zeolitic materials. For example, evaluated Fe(III)-, Al(III)-, and Mn(IV)-modified potassium clinoptilolite for simultaneous ammonium and phosphate recovery from treated urban wastewater. Despite the presence of various competing ions (Na^+^, K^+^, Mg^2+^, SO_4_^2−^, Cl^−^, NO_3_^−^), the modified zeolites showed high selectivity for ammonium via ion exchange and for phosphate via surface complexation. However, phosphate adsorption was more affected by Cl^−^, SO_4_^2−^, and NO_3_^−^, while ammonium uptake was interfered by cations such as Na^+^, Ca^2+^, Mg^2+^, as well as by the presence of organic matter adsorbed within the zeolitic matrix [12,70]. Similar findings were reported in synthetic wastewater systems, where phosphate and ammonium adsorption on Fe (III)-, Al (III)-, and Mn (IV)-modified zeolites was moderately reduced in the presence of competing anions (especially sulfate and bicarbonate). The interference was more pronounced for phosphate due to shared sorption mechanisms with these anions (e.g., ligand exchange); while ammonium uptake was also reduced by monovalent and divalent cations such as Na^+^ and Ca^2+^ [52,53,71].

These results are consistent with the diminished nutrient removal observed for CB–M in real wastewater, where the complex ionic matrix, including SO_4_^2−^, NO_3_^−^, Cl^−^, and HCO_3_^−^, likely contributes to competitive inhibition at the biochar surface. Nevertheless, CB–M maintained high phosphate selectivity, suggesting that strong chemisorptive interactions, such as inner-sphere complexation with metal (oxy)hydroxide phases, are less susceptible to interference from competing ions. These studies confirm that competitive ion effects are a critical factor in real-world applications and justify the lower performance observed in the CB–M system when moving from synthetic to real effluent matrices. Beyond ionic competition, other physicochemical factors such as high turbidity, cyanide content, ammonia nitrogen, and the presence of organic matter may alter the speciation of target ions or saturate the surface charge of biochar [58]. These findings reinforce the importance of evaluating nutrient adsorption under realistic operating conditions and highlight the need for future studies to perform controlled interference assays, both single-ion and multi-ion, to better understand the selectivity and robustness of CB–M in complex wastewater environments.

### 3.8. Comparative Efficiency of CB–M for Phosphate and Ammonium Removal

The performance of CB–M was compared with other biochar-based adsorbents reported in the literature for simultaneous phosphate and ammonium removal (Table 6). While most biochars have been evaluated for single-nutrient removal, studies addressing dual-nutrient recovery remain limited [72]. A comparative overview was performed highlighting differences in source material, modification strategies, and resulting adsorption capacities. The reported values represent maximum adsorption capacities under optimized experimental conditions, remarking that variations in operational parameters and wastewater composition may influence the performance. CB–M demonstrated superior phosphate adsorption capacity, ranging from 42.0 mg·g^−1^ to 72.0 mg·g^−1^ depending on temperature, while ammonium adsorption remained moderate (2.8–3.00 mg·g^−1^). These results indicate that CB–M interacts primarily with phosphate via chemisorption onto metal (oxy)hydroxide surfaces, whereas ammonium is removed through weaker physical interactions, such as electrostatic attraction and ion exchange.

To further contextualize the role of surface area in nutrient adsorption, it is useful to compare CB–M with other spent coffee ground-derived biochars reported in the literature. For example, bimetallic-modified spent coffee grounds biochar (Mg/Zr/CNBC) obtained via acid digestion followed by metal impregnation showed an increase in specific surface area from 1.3 to 145.9 m^2^·g^−1^ and a maximum phosphate adsorption capacity of 140.8 mg·g^−1^ [73]. Similarly, calcium-modified spent coffee grounds biochar increased its surface area from 0.47 to 72.9 m^2^·g^−1^, corresponding to a maximum phosphate adsorption capacity of 70.3 mg·g^−1^ [74], while Mg-modified spent coffee grounds biochar increased in surface area from 0.2 to 36.4 m^2^·g^−1^ with a phosphate adsorption capacity of 63.5 mg·g^−1^ [75]. These comparative findings highlight that although an increase in surface area generally improves adsorption performance, the relationship is not strictly linear with phosphate or ammonium uptake [45,64]. Rather, numerous studies emphasize that the presence of surface-bound metal (oxy)hydroxide species, such as those Fe, Zn, Mg, and Ca, plays a more critical role in improving phosphate adsorption. This enhancement is primarily achieved through chemisorption mechanisms, including inner-sphere complexation and ligand exchange. Therefore, in the context of CB–M, although the surface area increased from 3 to 22 m^2^·g^−1^ after modification, the key enhancement in adsorption performance is attributed primarily to the incorporation of Fe, Zn, and Mn (oxy)hydroxides phases. This reinforces the notion that surface chemistry, rather than surface area alone, is the dominant factor governing phosphate and ammonium adsorption on metal-modified biochars and validates the strategic functionalization approach used in this study.

Compared with biochars derived from soybean straw, rape straw, rice husk, sugarcane, or peanut shell, typically modified with magnesium, aluminum, or calcium, CB–M incorporates iron, zinc, and manganese, which are essential plant micronutrients. This provides CB–M with a dual benefit: high nutrient removal efficiency and environmental compatibility, making it suitable for reuse as a slow-release fertilizer with a minimal risk of secondary contamination, because other elements that can pollute soil are not introduced. Thereby it is an interesting option for closing the nutrient loop and promoting sustainable waste valorization.

Furthermore, CB–M functions effectively under near-neutral pH conditions, aligning with typical wastewater properties and reducing the need for pH adjustment, an operational over other biochar that requires stringent pH control. Although other modified biochars have shown higher ammonium, CB–M offers selectivity towards phosphate, multifunctionality, and compatibility with agricultural applications, supporting its use in circular economy strategies. This selectivity is particularly relevant for real wastewater treatment, where complex ion composition may impair overall adsorption performance. Consequently, CB–M represents a promising, sustainable alternative for integrated nutrient recovery and waste valorization.

**Table 6:** Comparison of phosphate and ammonium removal efficiencies of various biochars.

Biochar Source	Modification Strategy	Phosphate Adsorption Capacity (mg·g^−1^)	Ammonium Removal Capacity (mg·g^−1^)	Reference
Spent coffee grounds	CB–M biochar modified with Mn^2+^/Zn^2+^/Fe^3+^ (oxy)hydroxide nanoparticles	42.6	2.79	This study
		30.16 *	1.84 *	
Soybean straw	Impregnated with the prepared solutions MgCl_2_ and AlCl_3_	74.47	0.70	[76]
Rape straw (RS)	Red mud and rape Straw mixing	11.78	2.97	[76]
Rice husk biochar	Biochar supported Mg(OH)_2_/bentonite composite (PMRB)	125.36	58.20	[77]
Sugarcane crop harvest residue	MgO particle-impregnated	398	22	[78]
Peanut shell biochar	Mg-doped biochar/bentonite composite bead (SA-Mg@BC/BT)	132.2	39.5	[54]
Rice straw biochar	Calcium alginate-biochar composite at 300 °C (CA-MRB300)	31.38	1137.7	[55]
Biochar from the pyrolysis of sludge fermented	Biochar from the pyrolysis of sludge fermented with rusty scrap iron and reduced iron powder (RSI-RIP) at 600 °C-ES600	12.70	10.72	[79]
Crushed rice straws biochar	Immersion in MgCl_2_ solutions (HM-HP-HT) 2 M at 800 °C	5.52	4.40	[68]
Rice straw biochar	Modified by ferric chloride (Fe-HBC2)	22.98	28.10	[80]
Corn stalk biochar (BC)	Mg-modified biochar (MBC)	64.48	36.27	[81]

* Asterisked values indicate performance in real wastewater.

The maximum phosphate adsorption capacity of CB–M (up to 42.6 mg·g^−1^) is lower than that reported for some engineered adsorbents, such as Mn^2+^/Zn^2+^/Fe^3+^ (oxy)hydroxide layered double hydroxide (LDH) material [23]. CB–M offers notable advantages in terms of synthesis simplicity, sustainability, and practical applicability. LDH-based adsorbents often require precise co-precipitation protocols, the use of high-purity chemical reagents, and energy-intensive steps such as thermal aging, calcination, or pH-controlled crystallization. Furthermore, these materials typically operate optimally under alkaline conditions (pH > 8), which may limit their effectiveness in real wastewater matrices or when applied as soil amendments. Their structural stability is also known to degrade under acidic or neutral conditions, potentially compromising their long-term reusability. In contrast, CB–M provides several strategic advantages. It is derived from spent coffee grounds, an abundant and low-cost agro-industrial residue, and modified with readily available, low-toxicity metal salts (FeCl_3_, ZnCl_2_, MnCl_2_) under environmentally benign conditions. The synthesis is carried out at ambient temperature and near-neutral pH (~7) without the need for calcination, which facilitates the in situ precipitation and stable immobilization of metal (oxy)hydroxide phases on the porous biochar matrix. This method is not only energy-efficient and scalable but also minimizes chemical hazards. Additionally, the use of micronutrient metals (Fe, Zn, Mn) ensures that the spent biochar can be safely applied as a phosphorus-enriched soil amendment, contributing to sustainable agriculture. Biochar was, therefore, intentionally selected as a more sustainable and field-compatible carrier for metal (oxy)hydroxide immobilization. The observed selective phosphate uptake under near-neutral conditions further enhances the practical relevance of CB–M for integrated nutrient recovery in decentralized and low-resource treatment systems. Despite its relatively lower phosphate uptake capacity compared to some LDH systems, CB–M offers a compelling balance of cost-effectiveness, environmental compatibility, and operational simplicity, positioning it as a viable and scalable solution for phosphorus recovery and circular waste valorization.

Moreover, the economic viability of CB–M strengthens its potential for real-world application. Spent coffee grounds are an abundant waste product requiring only a structured collection strategy for consistent supply. The modifying agents, FeCl_3_, ZnCl_2_, and MnCl_2_, are low-cost, commercially available, and environmentally benign due to their roles as essential micronutrients in plant physiology. The synthesis is conducted under mild conditions (ambient temperature, atmospheric pressure, neutral pH), avoiding the need for specialized equipment or high energy input. Collectively, these attributes support the low-cost and scalable production of CB–M, particularly suited to decentralized wastewater treatment in rural or resource-limited regions, where simplicity, local material reuse, and environmental safety are essential.

## 4. Conclusions

This study demonstrates that the spent coffee ground-derived biochar, modified with Mn^2+^, Zn^2+^, and Fe^3+^ (oxy)hydroxide nanoparticles (CB–M) is a highly effective adsorbent for phosphate recovery from aqueous media. Structural characterization (XRD, SEM, FTIR) confirmed the successful metal incorporation, lattice distortion, and the development of hierarchical porosity, which increased surface area and enhanced reactive site density. Under near-neutral pH and ambient temperature conditions, CB–M achieved a maximum phosphate adsorption capacity of 42.0 mg·g^−1^, primarily via chemisorption onto metal (oxy)hydroxide sites. Ammonium removal was more modest (2.8 to 3.0 mg·g^−1^), governed by weaker ion exchange mechanisms. Over 70% of adsorbed phosphate was associated with Fe–Mn–Zn phases, supporting inner–sphere complexation and its potential for controlled nutrient release. CB–M exhibited good structural stability and partial irreversibility in phosphate retention, supporting its potential reuse as a slow–release phosphorus amendment Application to real municipal wastewater confirmed CB–M’s phosphate removal efficiency (<0.01 mg·L^−1^) and moderate ammonium uptake (1.84 mg·g^−1^), despite the presence of interfering anions. Although low nutrient desorption was observed, the potential leaching of incorporated metals, particularly Mn^2+^, warrants further assessment to ensure long-term environmental safety.

While the specific surface area of CB–M remains modest compared to highly engineered materials, its enhanced performance is driven by functionalization with metal (oxy)hydroxide groups, emphasizing the dominant role of surface chemistry over surface area in nutrient adsorption. CB–M shows high phosphate removal at neutral pH without pretreatment, offering dual-nutrient recovery and strong potential for sustainable wastewater treatment. Future work should enhance CB–M’s dual-nutrient capture by improving surface area and active site accessibility through advanced activation and impregnation strategies (e.g., template-assisted activation, post-pyrolysis etching, and multi-step metal impregnation). Finally, the economic and operational feasibility of CB–M is supported by its use of abundant, low-cost feedstock, environmentally benign metal precursors, and low-energy synthesis conditions. These features make CB–M an attractive, scalable, and sustainable solution for decentralized wastewater treatment, particularly in low-resource settings.

## Figures and Tables

**Table 1:** Chemical composition of biochar from spent coffee grounds (CB) and its metal-modified form (CB–M).

Components	CB	CB–M
	(%)	(%)
SiO_2_	2.4 ± 0.3	0.7 ± 0.4
P_2_O_5_	1.3 ± 0.1	2.5 ± 0.1
K_2_O	4.4 ± 0.0	1.3 ± 0.0
CaO	0.7 ± 0.0	2.2 ± 0.0
MnO	−	1.6 ± 0.0
Fe_2_O_3_	0.1 ± 0.0	25.3 ± 0.0
ZnO	−	63.9 ± 0.1

**Table 2:** Kinetic parameters of phosphate and ammonium adsorption onto metal-modified biochar from spent coffee grounds (CB–M).

Model	Kinetic Parameters		
		Phosphate	Ammonium
Pseudo-first order	q_e_ (mg∙g^−1^)	4.98	0.07
	k_1_ (h^−1^)	3.03	0.27
	R^2^	0.98	0.90
Pseudo-second order	q_e_ (mg∙g^−1^)	6.38	0.12
	k_2_ (h^−1^)	1.95	43.00
	R^2^	1.00	1.00
Intraparticle diffusion	k_1_ (h^−1^)	10.57	5 × 10^−1^
	R^2^	0.99	0.99
	k_2_ (h^−1^)	2.66	3.01 × 10^−2^
	R^2^	0.96	0.97
Film diffusion	k_F_ (h^−1^)	0.05	4.54 × 10^−3^
	R^2^	0.98	0.90
Particle diffusion	k_P_ (h^−1^)	0.02	1.99 × 10^−3^
	R^2^	1.00	0.94

**Table 3:** Isotherm parameters for phosphate and ammonium adsorption onto metal-modified biochar from spent coffee grounds (CB–M).

Model		T = 293.15 K		T = 299.15 K		T = 306.15 K	
		PO_4_^3−^	NH_4_^+^	PO_4_^3−^	NH_4_^+^	PO_4_^3−^	NH_4_^+^
Langmuir	q_m_ (mg∙g^−1^)	42.6	2.79	57.8	2.84	72.0	2.95
	k_L_ (L∙g^−1^)	1.2 × 10^–2^	2.4 × 10^–2^	7.5 × 10^–3^	2.7 × 10^–2^	6.7 × 10^–3^	2.8 × 10^–2^
	R^2^	0.98	0.60	0.95	0.56	0.92	0.80
Freundlich	k_F_ (mg∙g^−1^)	5.4	0.13	5.3	0.09	5.6	0.16
	1/n	0.3	0.62	0.3	0.74	0.3	0.59
	R^2^	0.83	0.90	0.85	0.87	0.86	0.95

**Table 4:** Thermodynamic parameters for phosphate and ammonium adsorption onto metal-modified biochar (CB–M).

Temperature	ln k_c_		R^2^	ΔG°		ΔS°		ΔH°	
(K)	PO_4_^3−^	NH_4_^+^		PO_4_^3−^	NH_4_^+^	PO_4_^3−^	NH_4_^+^	PO_4_^3−^	NH_4_^+^
				(kJ·mol^−1^)		(kJ·mol^−1^·K^−1^)		(kJ·mol^−1^)	
293.15	61.0	23.6	0.84	−148.8	−57.5	−4	1.02	1450	−241
299.15	39.2	27.4		−97.6	−68.1				
306.15	35.4	27.9		−90.1	−70.9				

**Table 5:** Fractionation of phosphate from CB–M after adsorption.

Fractions Type	Parameters	
Total adsorbed PO_4_^3−^	q_e_ (mg∙g^−1^)	5.6
PO_4_^3−^ labile fraction	%	17.1
	q_e_ (mg∙g^−1^)	0.37
PO_4_^3−^ Fe-Mn-Zn bound	%	72.1
	q_e_ (mg∙g^−1^)	1.6
PO_4_^3−^ Na-Mg-Ca bound	%	6.3
	q_e_ (mg∙g^−1^)	0.1
PO_4_^3−^ Residual	%	2.7
	q_e_ (mg∙g^−1^)	0.1

## Data Availability

The original contributions presented in this study are included in the article and Supplementary Material. Further inquiries can be directed to the corresponding author.

## Supplementary Materials

The following supporting information can be downloaded at: https://www.mdpi.com/article/10.3390/nano15110849/s1, Table S1. Physicochemical and microbiological properties of the Wastewater sample from Wastewater Treatment Plant of Loja City. Figure S1. Precipitation and speciation behavior of Fe^3+^, Zn^2+^, and Mn^2+^ as a function of pH (generated by Medusa).
