# Supplementary material for: Trimetallic Fe-Zn-Mn (Oxy)Hydroxide-Enhanced Coffee Biochar for Simultaneous Phosphate and Ammonium Recovery and Recycling
Source: Nanomaterials (Basel). 2025 Jun 2;15(11):849. doi: 10.3390/nano15110849 (PMC12157899; doi:10.3390/nano15110849)
Supplement: Supplementary file 1 [file nanomaterials-15-00849-s001.zip › nanomaterials-3631424-supplementary/nanomaterials-3631424-supplementary.docx]

Trimetallic Fe–Zn–Mn (Oxy)Hydroxide-Enhanced Coffee Biochar for Simultaneous Phosphate and Ammonium Recovery and Recycling

Diana Guaya ^1,^*, Jhuliana Campoverde ^1^, Camilo Piedra ^2^ and Alexis Debut ^3^

^1^ Departamento de Química, Universidad Técnica Particular de Loja, Loja 110107, Ecuador

^2^ Escuela de Ingeniería Química, Universidad Técnica Particular de Loja, Loja 110107, Ecuador

^3^ Centro de Nanociencia Nanotecnología, Universidad de las Fuerzas Armadas ESPE, Sangolquí 171103

***** Correspondence: deguaya@utpl.edu.ec

**Table S1.** Physicochemical and microbiological properties of the Wastewater sample from Wastewater Treatment Plant of Loja City.


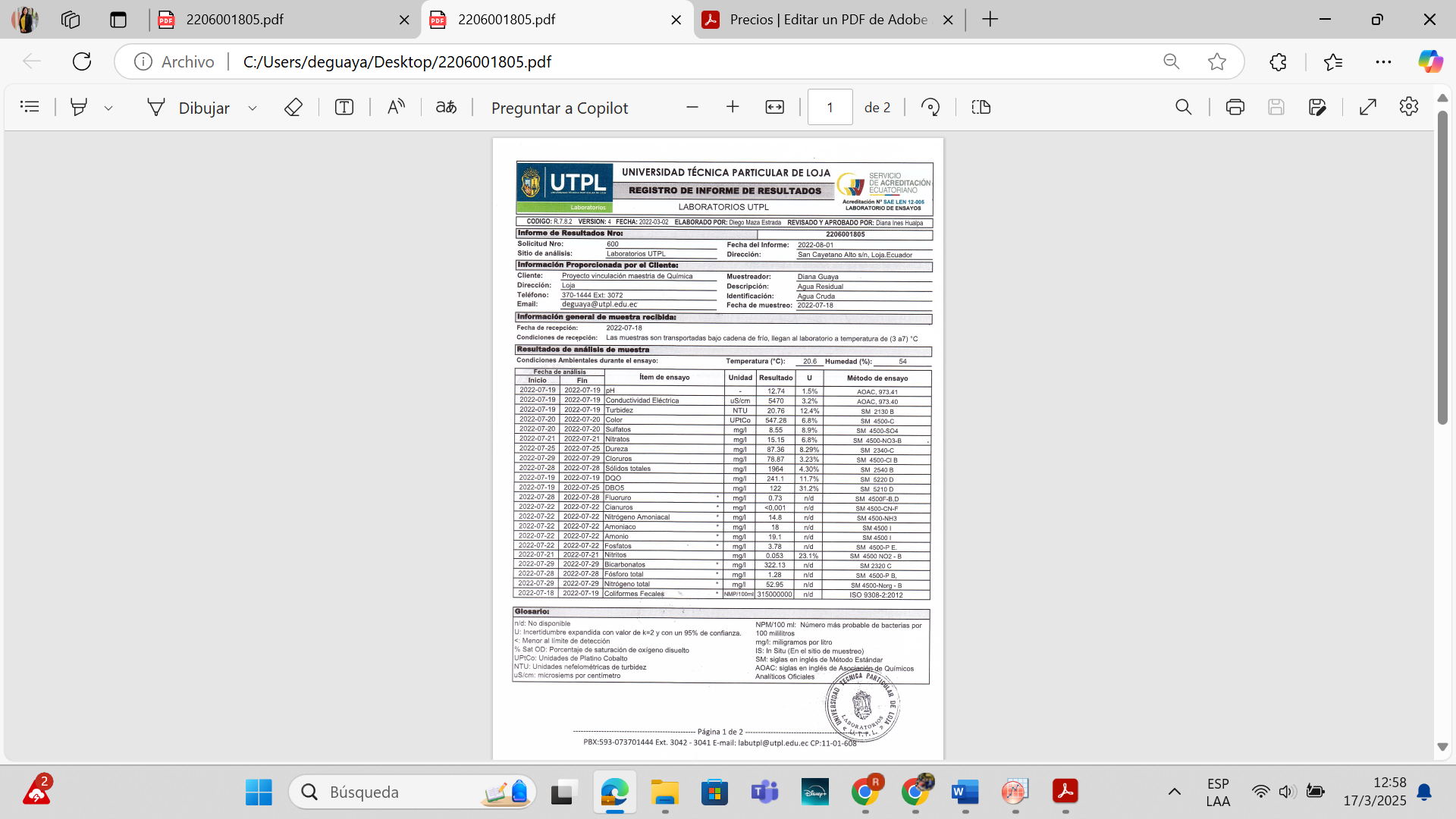


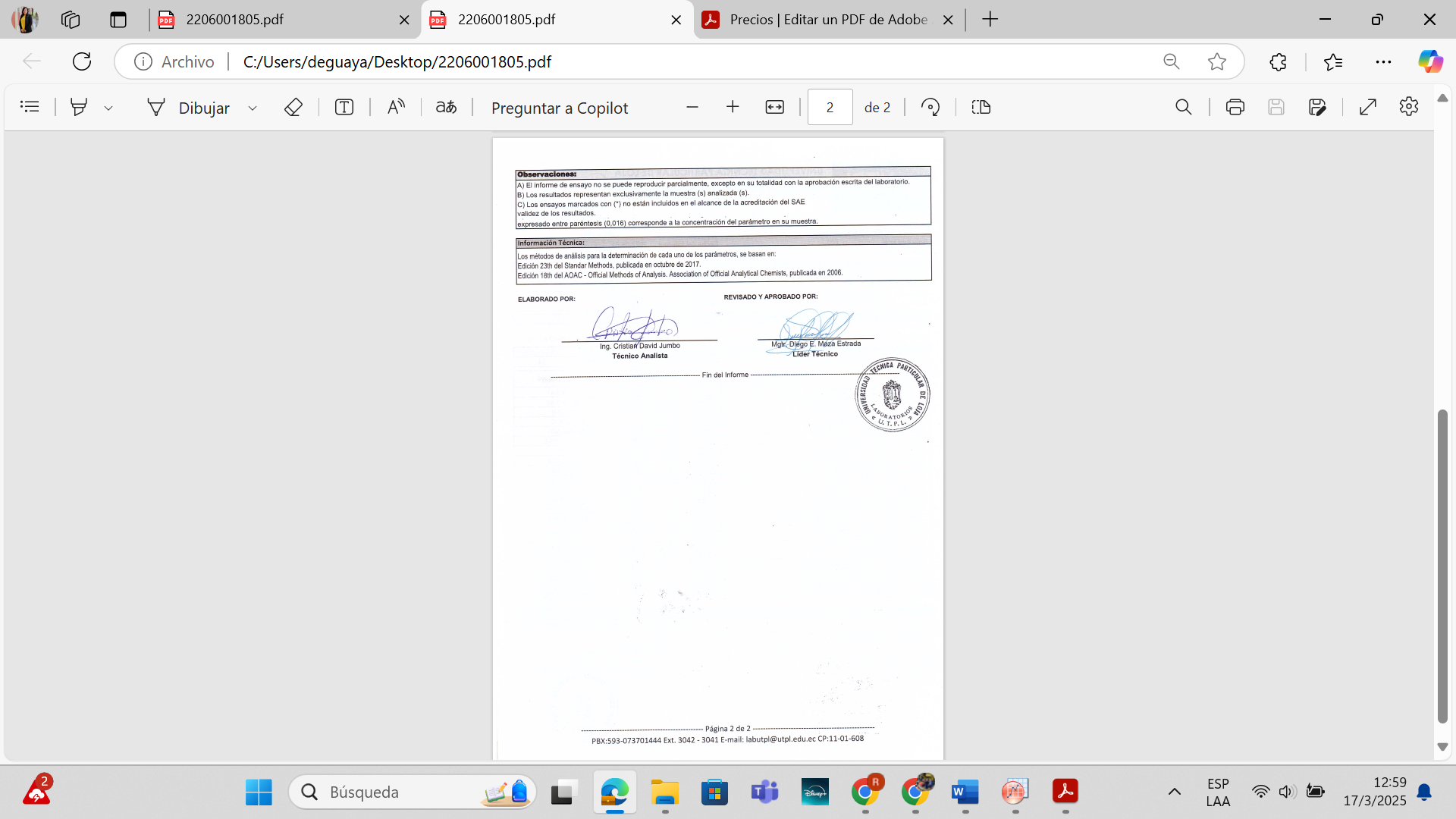


**Figure S1.** Precipitation and speciation behavior of Fe³⁺, Zn²⁺, and Mn²⁺ as a function of pH (generated by Medusa).


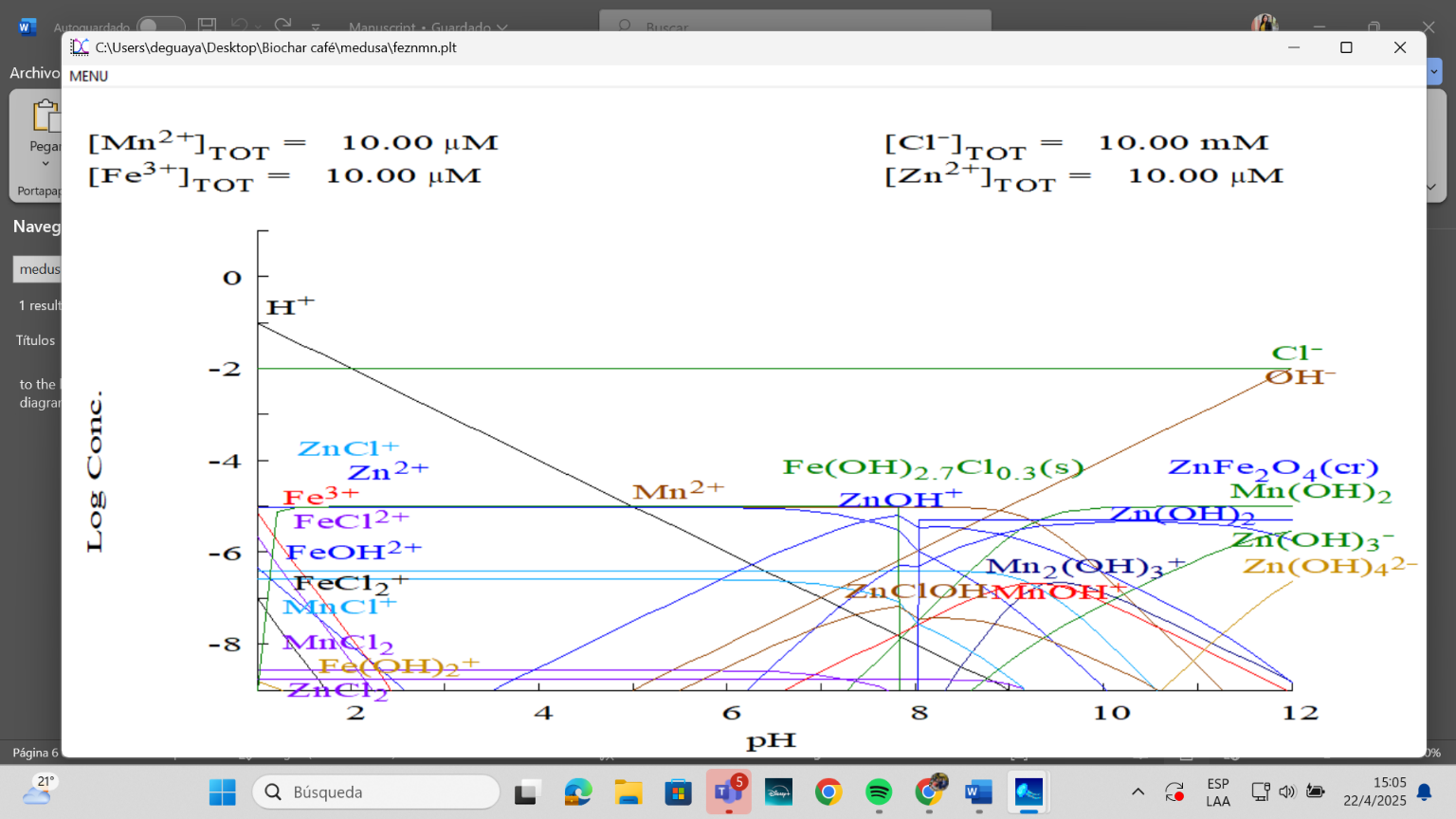


**pH= 7**

**Predominant aqueous species (Fe³⁺, Zn²⁺, Mn²⁺) at experimental conditions**
