# Supplementary material for: Trimetallic Fe-Zn-Mn (Oxy)Hydroxide-Enhanced Coffee Biochar for Simultaneous Phosphate and Ammonium Recovery and Recycling
Source: Nanomaterials (Basel). 2025 Jun 2;15(11):849. doi: 10.3390/nano15110849 (PMC12157899; doi:10.3390/nano15110849)
Supplement: Supplementary file 1 [file nanomaterials-15-00849-s001.zip › nanomaterials-3631424-supplementary/nanomaterials-3631424-supplementary.pdf]

Trimetallic Fe–Zn–Mn (Oxy)Hydroxide-Enhanced Coffee Biochar for Simultaneous Phosphate and Ammonium Recovery and Recycling

Diana Guaya [1,*], Jhuliana Campoverde [1], Camilo Piedra [2] and Alexis Debut [3]

[1] Departamento de Química, Universidad Técnica Particular de Loja, Loja 110107, Ecuador
[2] Escuela de Ingeniería Química, Universidad Técnica Particular de Loja, Loja 110107, Ecuador
[3] Centro de Nanociencia Nanotecnología, Universidad de las Fuerzas Armadas ESPE, Sangolquí 171103
* Correspondence: deguaya@utpl.edu.ec

Table S1. Physicochemical and microbiological properties of the Wastewater sample from Wastewater Treatment Plant of Loja City.

UNIVERSIDAD TÉCNICA PARTICULAR DE LOJA

REGISTRO DE INFORME DE RESULTADOS

LABORATORIOS UTPL

SERVICIO DE ACREDITACIÓN ECUATORIANO

Acreditación N° SAE LEN 12-005 LABORATORIO DE ENSAYOS

CODIGO: R.7.8.2 **VERSION:** 4 **FECHA:** 2022-03-02 **ELABORADO POR:** Diego Maza Estrada **REVISADO Y APROBADO POR:** Diana Ines Hualpa

Informe de Resultados Nro: 2206001805

Solicitud Nro: 600
Fecha del Informe: 2022-08-01
Sitio de análisis: Laboratorios UTPL
Dirección: San Cayetano Alto s/n, Loja.Ecuador

Información Proporcionada por el Cliente:

Cliente: Proyecto vinculación maestria de Química
Dirección: Loja
Teléfono: 370-1444 Ext: 3072
Email: deguaya@utpl.edu.ec
Muestreador: Diana Guaya
Descripción: Agua Residual
Identificación: Agua Cruda
Fecha de muestreo: 2022-07-18

Información general de muestra recibida:

Fecha de recepción: 2022-07-18
Condiciones de recepción: Las muestras son transportadas bajo cadena de frio, llegan al laboratorio a temperatura de (3 a7) °C

Resultados de análisis de muestra

Condiciones Ambientales durante el ensayo: **Temperatura (°C):** 20.6 **Humedad (%):** 54

Fecha de análisis		Ítem de ensayo		Unidad	Resultado	U	Método de ensayo
Inicio	Fin						
2022-07-19	2022-07-19	pH		-	12.74	1.5%	AOAC, 973.41
2022-07-19	2022-07-19	Conductividad Eléctrica		uS/cm	5470	3.2%	AOAC, 973.40
2022-07-19	2022-07-19	Turbidez		NTU	20.76	12.4%	SM 2130 B
2022-07-20	2022-07-20	Color		UPtCo	547.28	6.8%	SM 4500-C
2022-07-20	2022-07-20	Sulfatos		mg/l	8.55	8.9%	SM 4500-SO4
2022-07-21	2022-07-21	Nitratos		mg/l	15.15	6.8%	SM 4500-NO3-B
2022-07-25	2022-07-25	Dureza		mg/l	87.36	8.29%	SM 2340-C
2022-07-29	2022-07-29	Cloruros		mg/l	78.87	3.23%	SM 4500-Cl B
2022-07-28	2022-07-28	Sólidos totales		mg/l	1964	4.30%	SM 2540 B
2022-07-19	2022-07-19	DQO		mg/l	241.1	11.7%	SM 5220 D
2022-07-19	2022-07-25	DBO5		mg/l	122	31.2%	SM 5210 D
2022-07-28	2022-07-28	Fluoruro	*	mg/l	0.73	n/d	SM 4500F-B,D
2022-07-22	2022-07-22	Cianuros	*	mg/l	<0,001	n/d	SM 4500-CN-F
2022-07-22	2022-07-22	Nitrógeno Amoniacal	*	mg/l	14.8	n/d	SM 4500-NH3
2022-07-22	2022-07-22	Amoniaco	*	mg/l	18	n/d	SM 4500 I
2022-07-22	2022-07-22	Amonio	*	mg/l	19.1	n/d	SM 4500 I
2022-07-22	2022-07-22	Fosfatos	*	mg/l	3.78	n/d	SM 4500-P E.
2022-07-21	2022-07-21	Nitritos		mg/l	0.053	23.1%	SM 4500 NO2 - B
2022-07-29	2022-07-29	Bicarbonatos	*	mg/l	322.13	n/d	SM 2320 C
2022-07-28	2022-07-28	Fósforo total	*	mg/l	1.28	n/d	SM 4500-P B,
2022-07-29	2022-07-29	Nitrógeno total	*	mg/l	52.95	n/d	SM 4500-Norg - B
2022-07-18	2022-07-19	Coliformes Fecales	*	NMP/100ml	315000000	n/d	ISO 9308-2:2012

Glosario:

n/d: No disponible
U: Incertidumbre expandida con valor de k=2 y con un 95% de confianza.
<: Menor al límite de detección
% Sat OD: Porcentaje de saturación de oxígeno disuelto
UPtCo: Unidades de Platino Cobalto
NTU: Unidades nefelométricas de turbidez
uS/cm: microsiems por centímetro
NPM/100 ml: Número más probable de bacterias por 100 mililitros
mg/l: miligramos por litro
IS: In Situ (En el sitio de muestreo)
SM: siglas en inglés de Método Estándar
AOAC: siglas en inglés de Asociación de Químicos Analíticos Oficiales

UNIVERSIDAD TÉCNICA PARTICULAR DE LOJA LABORATORIOS U.T.P.L.

PBX:593-073701444 Ext. 3042 - 3041 E-mail: labutpl@utpl.edu.ec CP:11-01-608

Observaciones:

A) El informe de ensayo no se puede reproducir parcialmente, excepto en su totalidad con la aprobación escrita del laboratorio.
B) Los resultados representan exclusivamente la muestra (s) analizada (s).
C) Los ensayos marcados con (*) no están incluidos en el alcance de la acreditación del SAE
validez de los resultados.
expresado entre paréntesis (0,016) corresponde a la concentración del parámetro en su muestra.

Información Técnica:

Los métodos de análisis para la determinación de cada uno de los parámetros, se basan en:
Edición 23th del Standar Methods, publicada en octubre de 2017.
Edición 18th del AOAC - Official Methods of Analysis. Association of Official Analytical Chemists, publicada en 2006.

ELABORADO POR:	REVISADO Y APROBADO POR:
Ing. Cristian David Jumbo	Mgtr. Diego E. Maza Estrada
**Técnico Analista**	**Líder Técnico**

UNIVERSIDAD TECNICA PARTICULAR DE LOJA LABORATORIOS « U. T. P. L. »

------------ Fin del Informe ------------

PBX:593-073701444 Ext. 3042 - 3041 E-mail: labutpl@utpl.edu.ec CP:11-01-608

Figure S1. Precipitation and speciation behavior of Fe^{3+}, Zn^{2+}, and Mn^{2+} as a function of pH (generated by Medusa).

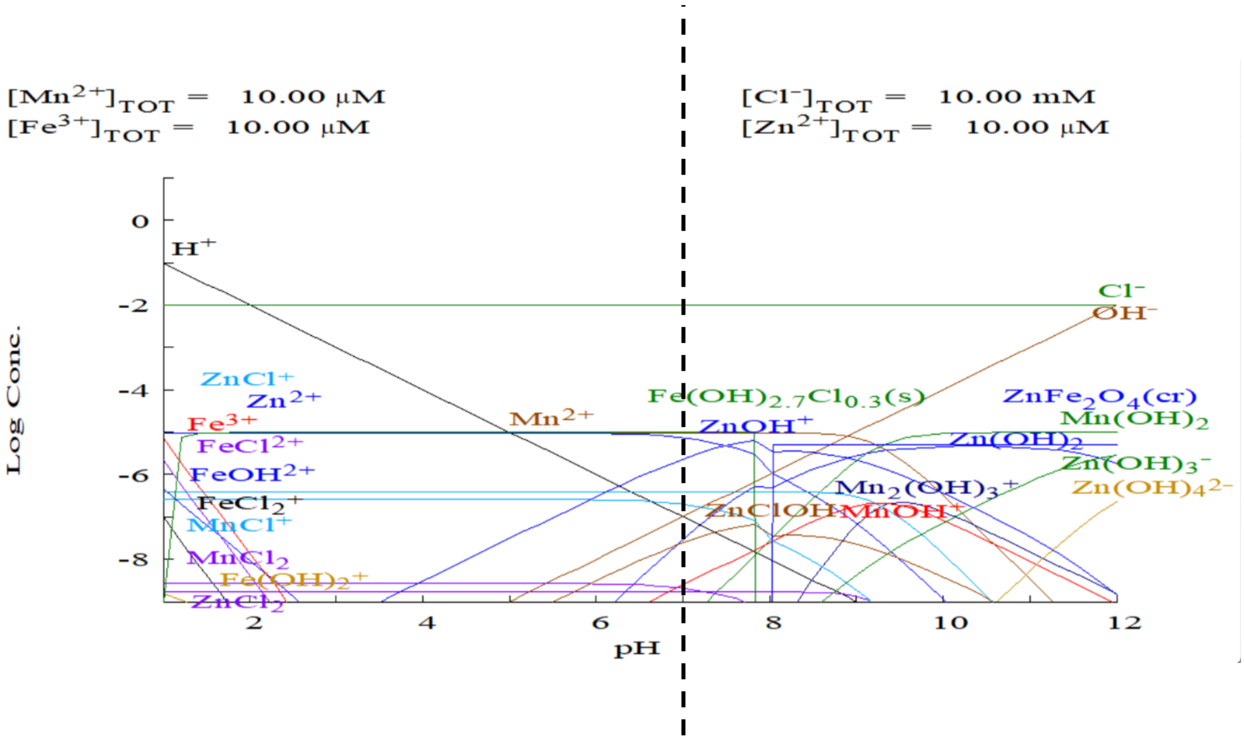

pH= 7

Predominant aqueous species (Fe^{3+}, Zn^{2+}, Mn^{2+}) at experimental conditions
